# SOSORT Award Winner 2015: a multicentre study comparing the SPoRT and ART braces effectiveness according to the SOSORT-SRS recommendations

**DOI:** 10.1186/s13013-015-0049-4

**Published:** 2015-08-11

**Authors:** Fabio Zaina, Jean Claude de Mauroy, Sabrina Donzelli, Stefano Negrini

**Affiliations:** ISICO, Italian Scientific Spine Institute, Milan, Italy; Clinique du Parc, Lyon, France; Brescia University, Brescia, Italy; Fondazione Don Gnocchi, Brescia, Italy

## Abstract

**Background:**

Data comparing different braces for adolescent idiopathic scoliosis (AIS) are scant. The SRS criteria represent some guidelines for comparing results from different studies, but controlled studies are much more reliable. Recently, super-rigid braces have been introduced in clinical practice with the aim of replacing Risser and EDF casts. The aim of the present study is to compare the short-term radiographic results of two super-rigid braces, the ART and the SPORT (Sforzesco) brace.

**Methods:**

A group of consecutive patients with Cobb >40°, Risser 0–4, age >10 treated with the ART brace for 6 months were matched with a group of similar patients taken from a prospective database of patients treated with the Sforzesco brace. Patients were matched according to Cobb severity, pattern and localization of the curve.

All patients had a full-time brace prescription (23–24 hours per day) and an indication to perform scoliosis-specific exercises and were assessed radiographically both immediately in the brace and after 6 months of treatment out of brace. Curves were analyzed according to the pattern and localization taking into consideration both the in-brace correction and the 6-month out-of-brace results.

Statistical analysis: *t*-test, ANOVA, linear regression, alpha set at 0.05.

**Results:**

Twenty-six patients were included in the ART brace group, and 26 in the Sforzesco brace group. At baseline, no differences were noted for gender (3 males for each group), age (14.1 ± 0.3 for ART vs 13.9 ± 0.3 for Sforzesco), ATR (11.8 ± 3.2 vs 11.5 ± 4.2 for thoracic curves and 7.8 ± 4.0 vs 7.1 ± 6.1 for lumbar/thoracolumbar), Cobb angle (44.8 ± 2 vs 45.5 ± 2 for thoracic; 43.8 ± 2 vs 46.0 ± 2 for lumbar/thoracolumbar) or Risser sign (median 2 for both groups).

The in-brace correction was slightly better for the ART brace, but didn’t reach statistical significance (24.3 ± 8.5 vs 28.0 ± 6.8 for thoracic; 23.7 ± 10.4 vs 29.9 ± 4.2 for lumbar/thoracolumbar). At 6 months, results were similar both for thoracic (34.4 ± 10.4 vs34.8 ± 6.8) and for lumbar/thoracolumbar (32.8 ± 10.8 vs 36.6 ± 5.2). Also, with regard to the pattern, results were similar for double major and for thoracic, while there were not enough data for single lumbar to make a comparison.

No differences for ATR were found (7.8 ± 3.2 vs 8.6 ± 2.9 for thoracic; 4.3 ± 3.4 vs 4.3 ± 3.7 for lumbar/thoracolumbar).

**Conclusion:**

These two super-rigid braces showed similar short-term results, despite the better in-brace correction for lumbar curves shown by the ART brace. According to our data, the asymmetric design showed results similar to the symmetric one. After these preliminary data, further studies are needed to check end growth results and the impact of compliance, rigidity of curve, exercise and assessing quality of life.

**Electronic supplementary material:**

The online version of this article (doi:10.1186/s13013-015-0049-4) contains supplementary material, which is available to authorized users.

## Introduction

In the last two years, one randomized clinical trial (RCT) and one prospective controlled study with a randomized arm confirmed the efficacy of bracing in changing the natural history of scoliosis and dramatically reducing the need for surgery [1, 2]. Despite relying on similar biomechanical principles [3], today braces are quite different in design, and possibly in terms of efficacy [4]. This can depend on building differences, but also on managing protocols [5]. Nevertheless, literature is scant in terms of direct comparisons among different braces, and many studies are not even controlled [6]. For these reasons, in order to try to understand what the differences were among different braces, the Scoliosis Research Society created some criteria for comparing braces [7]. The first comparisons showed very different effects, with braces sometimes being highly ineffective [8], to the extent that their results have been considered just natural history [9], while others showed pretty good results [10, 11]. After this first attempt to induce researchers to work along a common path, new criteria were developed, in order to study braces and other conservative treatments in a wider range of scoliosis entity and age [12].

One of the main aims of conservative treatment is to avoid surgery [1, 13]. Casts have been shown to be very effective [14], although today they are mainly used for juvenile and infantile scoliosis [15, 16]. Recently a new generation of braces has been developed, so-called “super-rigid” braces. This characteristic of improved rigidity comes from being made of two big pieces of polycarbonate material, connected by an aluminum bar to allow for opening by the patient [17]. The first of this series was the Sforzesco brace [17], created in 2005 in Milan, and it showed immediate results that were better than the Lyon brace and similar to the Risser cast, with a better preservation of the sagittal balance of the spine [18–20]. The Sforzesco brace also showed itself to be capable of improving surgical scoliosis in more than 50 % of cases [20]. More recently, in 2013, the ART brace was created in Lyon, with the aim of replacing the EDF cast and the Lyon brace [21], and in this regard the first short-term results were positive [22].

Both these braces share a highly rigid material, being made in polycarbonate, while they differ in the envelope shape, with the Sforzesco being externally almost symmetric with expansion room inside, while the ART is totally asymmetric and built in a hyper-corrected posture. As they have some similarities but also many differences, it would be really interesting to know which one works better. For this reason, we designed this retrospective multicentre matched case–control study to compare the in-brace correction and the very short-term results at 6 months of the SPoRT (Sforzesco) brace and ART brace in a group of Adolescent Idiopathic Scoliosis (AIS) patients.

## Methods

### Setting

Two outpatient tertiary referral facilities specialized in scoliosis conservative treatment.

### Design

This is a study with a multicentre matched case–control design nested in two prospective databases including all the braced AIS patients at the two participating centers. These databases include all the super-rigid braces produced since their inception by the two groups who developed the concepts of the SPORT (Sforzesco) and ART braces. These databases include 1758 (from brace development in 2005 to September 2014) and 302 (from brace development in 2012 to September 2014) braced patients, respectively. The characteristics of patients included in the two databases are reported in Table 1.

**Table 1:** Characteristics of the two databases

Data base		Sforzesco	ART	Alpha
Total braced patients		1758	302	
Gender	Females	83.1 %	82.8 %	NS
	Males	16.9 %	17.2 %	
Cobb degrees	Below 20°	1.6 %	3.4 %	*P* < 0.05
	20-29°	12.0 %	42.7 %	
	20-39°	35.6 %	32.9 %	
	40° or more	50.8 %	20.9 %	
European Risser	0	27.2 %	36.7 %	*P* < 0.05
	1	13.8 %	20.8 %	
	2	18.5 %	10.1 %	
	3	24.7 %	9.2 %	
	4 or more	15.9 %	23.1 %	
Age	Below 10	3.2 %	3.3 %	NS
	10-12	29.2 %	21.5 %	
	13-14	39.1 %	41.4 %	
	15 or more	28.5 %	33.8 %	
Curve topography	Single thoracic	14.1 %	37.3 %	*P* < 0.05
	Single lumbar/thoracolumbar	10.2 %	27.0 %	
	Double thoracic/thoracolumbar or lumbar	67.9 %	35.7 %	
	Others	7.6 %	-	

### Participants

To compare the two databases, which appeared to be totally different (Table 1), we searched in the ART brace database all patients according to the following inclusion criteria: curves larger than 40°, Risser 0–4, age >10, treated for 6 months, immediate in-brace radiographs and 6 months out-of-brace radiographs available. This choice was made because in the Sforzesco brace database below the threshold of 40° other braces were prescribed too; consequently not all patients treated were included, but only the worst cases. This group was matched to a similar group of patients from the Sforzesco brace database according to Cobb severity, pattern and localization of the curve, age, ATR and sex.

### Treatment protocol

**Fig. 1:**
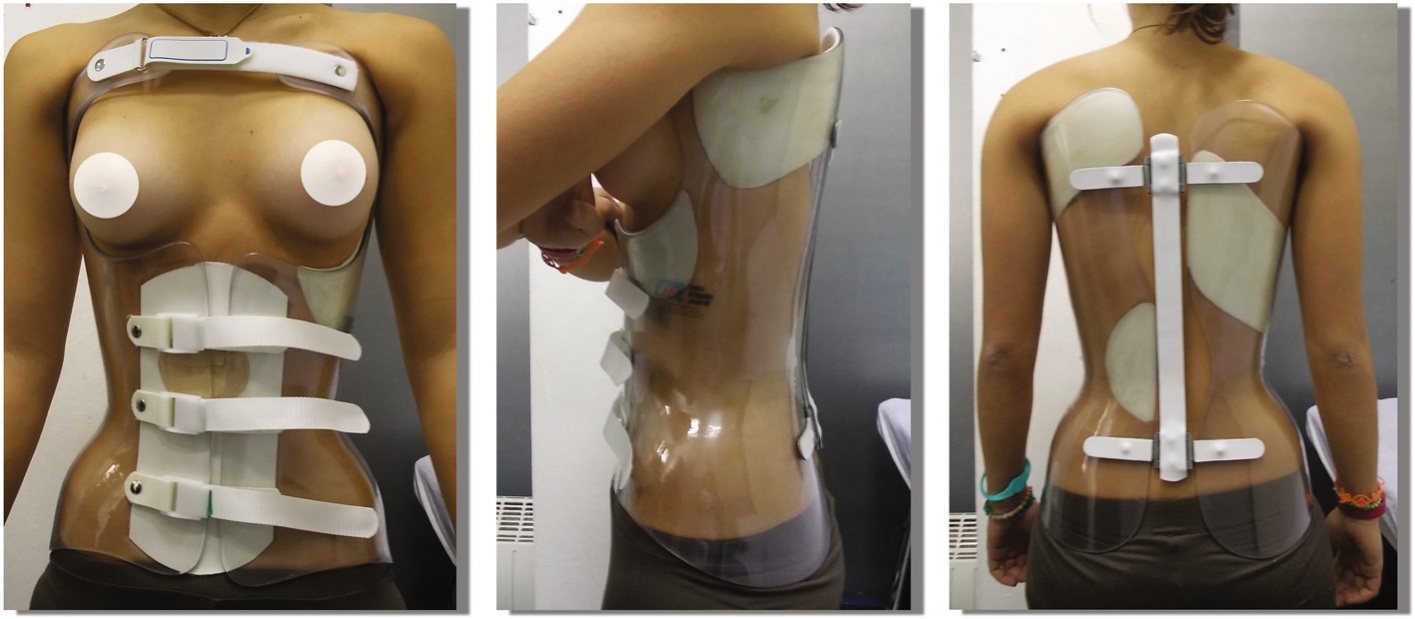
The Sforzesco (SPoRT) brace

All patients from both groups had a full-time brace prescription (23–24 hours per day) and indications to perform scoliosis-specific exercises. All patients were followed up according to SOSORT management criteria [5]. As previously described [17], the Sforzesco brace is constructed with rigid polycarbonate, in two pieces, connected posteriorly at the midline by a vertical aluminum bar and anteriorly by a closure over the breast and below is made of soft inelastic bands (Fig. 1). While the brace appears to be in full contact, in reality, due to its symmetry and according to the theoretical body shape the patient would have without scoliosis, it provides space over depressions and pushes over pathological elevations. The most relevant results of the Sforzesco brace are related to patients with large curves exceeding 45° but refusing surgical treatment, who improved in more that 50 % of cases [20], and comparisons with the Risser casts [19].

**Fig. 2:**
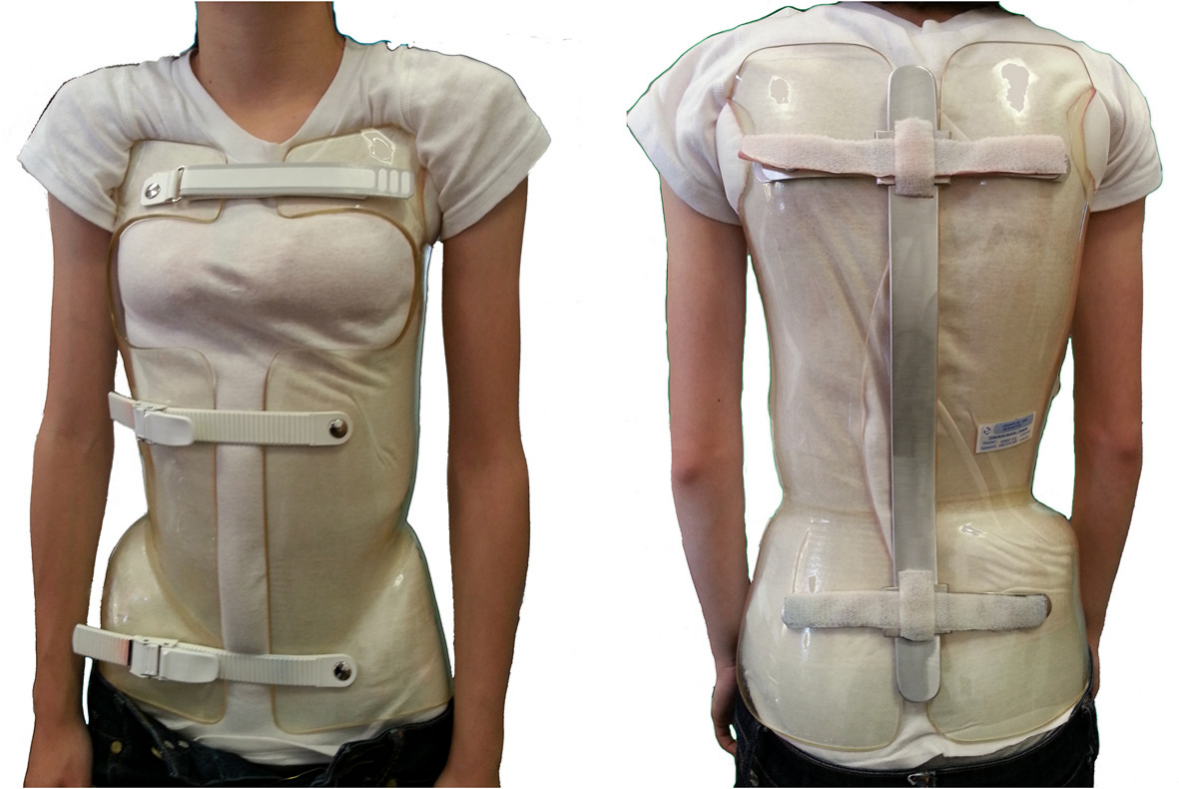
The ART brace

The ART brace (acronym for Asymmetrical, Rigid, Torsion brace), which has been described elsewhere [22], is also constructed with two rigid asymmetrical lateral pieces of polycarbonate, connected posteriorly at the midline by a duraluminum bar like the historical Lyon brace. All metal parts are similar to those of the Lyon brace (Fig. 2). Both the anterior and lower ratcheting buckles are rigid, and the upper third is Velcro. The brace is not in complete contact with the body: there is an expansion room in the concavity which is there to allow room for the body’s expansion during inhalation. It’s been applied in clinical practice since 2013, so the results published so far are related to the in-brace correction, which was quite good in all planes [22].

### Evaluations

Patients were assessed radiographically both in brace and out of brace after 6 months of treatment. In-brace radiographies were performed immediately for the ART brace, and after 1 month of brace wearing for the Sforzesco brace group. Curves were analyzed according to the pattern and localization taking into consideration both the in-brace correction and the 6-month results out of brace. We also measured the ATR (angle of trunk rotation); this is a clinical measurement of the hump made using the Bunnell scoliometer while the patient is bent forward performing the Adams test [23]. For the Risser sign, we used the European (French) version, which divides the excursion of the apophysis into thirds, with Stage 4 representing complete ossification and initiation of apophyseal fusion. The United States Risser staging system instead divides the excursion of the apophysis into quarters of the iliac crest beginning anterolaterally and progressing posteromedially [24].

We made no sample size calculation, since we had no data to rely on for such a comparison. Moreover, as the ART brace has been developed very recently, we included all the patients available. For statistical analysis we used ANOVA and a *t*-test; a linear regression model was applied to control for ATR, age and Risser. Alpha was set at 0.05.

### Ethics

This study respected the Helsinki Declaration on the testing of human subjects, and written informed consent was collected.

## Results

Twenty-six patients were included in the ART brace group, and 26 in the Sforzesco brace group. At baseline no differences were noted for gender, age, Risser sign, Cobb angle, ATR and time to first follow-up (Tables 2 and 3). Both groups scored 43 out of 44 on the “Standards of management of idiopathic scoliosis with corrective braces in everyday clinics and in clinical research” questionnaire (Additional files 1 and 2) [5].

**Table 2:** Baseline characteristics of the study population

	ART	Sforzesco	P
Number	26	26	NS
Males/females ratio	11.54 %	11.54 %	NS
Age (years)	14.1 ± 0.3	13.9 ± 0.3	NS
Risser Sign	2.22 ± 0.31	1.78 ± 0.32	NS
Cobb Angle thoracic (degrees)	44.8 ± 2	45.5 ± 2	NS
Cobb Angle lumbar/thoracolumbar (degrees)	43.8 ± 2	46.0 ± 2	NS
ATR thoracic (degrees)	11.8 ± 3.2	11.5 ± 4.2	NS
ATR lumbar/thoracolumbar (degrees)	7.8 ± 4.0	7.1 ± 6.1	NS
Time to first follow-up (months)	6	6	NS

**Table 3:** Baseline subgrouping

Data base		Sforzesco	ART
Total braced patients		26	26
Cobb degrees	40°-45°	53.85 %	57.69 %
	46°-50°	30.77 %	34.62 %
	>50°	15.38 %	7.69 %
European Risser	0-2	65.38 %	50.00 %
	3	26.92 %	15.38 %
	4>	7.69 %	34.63 %
Age	10-12	42.30 %	15.38 %
	13-14	34.62 %	46.15 %
	14 or more	23.08 %	38.46 %
Curve topography	Single thoracic	50.00 %	42.31 %
	Double thoracic/thoracolumbar or lumbar	50.00	57.69
	Others	%	%

**Fig. 3:**
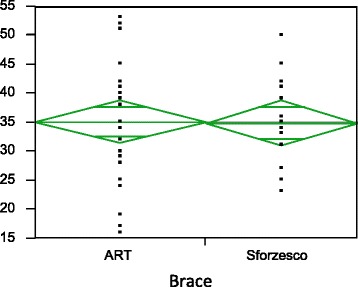
Six months results for Thoracic curves, *p* = NS

**Fig. 4:**
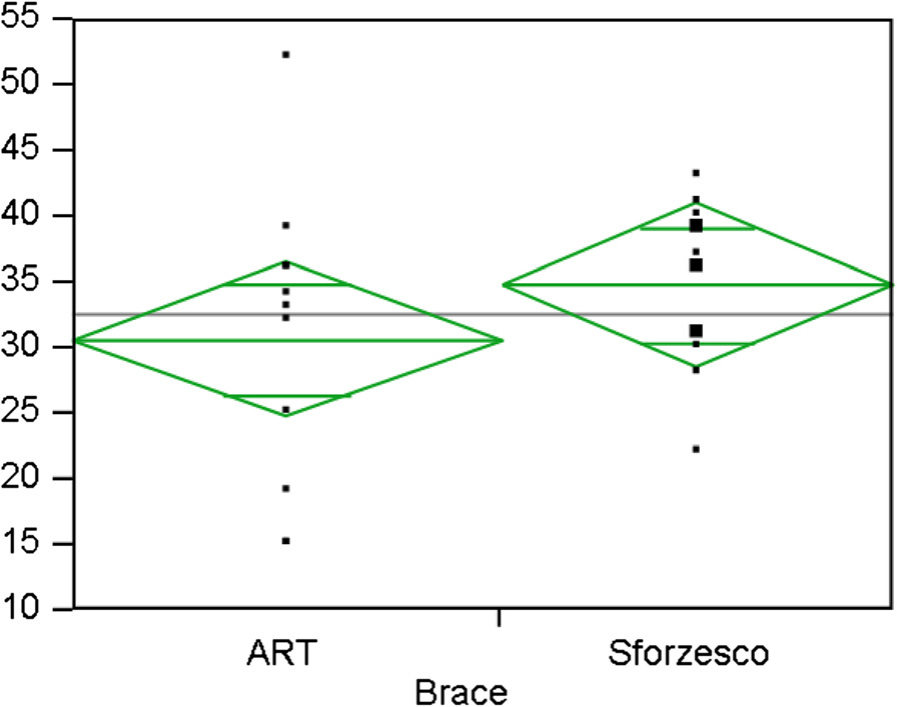
Six months results for Lumbar/thoracolumbar curves, *p* = NS

The in-brace correction was slightly better for the ART brace, but didn’t reach statistical significance (24.3 ± 8.5 vs 28.0 ± 6.8 for thoracic; 23.7 ± 10.4 vs 29.9 ± 4.2 for lumbar/thoracolumbar). At 6 months (Figs. 3 and 4), results were similar both for thoracic (34.4 ± 10.4 vs34.8 ± 6.8) and for lumbar/thoracolumbar (32.8 ± 10.8 vs 36.6 ± 5.2). Also, with regard to the pattern, results were similar for double major and for thoracic, while data for single lumbar were not enough to make a comparison. In the whole population and both groups, improvements were statistically significant from start to in-brace correction and to 6-month follow-up without brace. We found a loss of correction between in brace and out of brace for all curve patterns.

No differences for ATR after treatment were found (7.8 ± 3.2 vs 8.6 ± 2.9 for thoracic; 4.3 ± 3.4 vs 4.3 ± 3.7 for lumbar/thoracolumbar), while the improvement was statistically significant in both groups and for all locations.

## Discussion

This is the first multicenter case–control study comparing the first two super-rigid braces recently developed with the aim of replacing/avoiding casting [17, 19]. According to our results, there are no differences in the in-brace results nor in the short-term 6 months out-of-brace results. Apparently the design was more or less asymmetrical and did not cause real differences. The slight difference in favor of the ART brace could be confirmed or denied by a wider sample, but in any case was not clinically significant.

Despite relying on similar biomechanical principles [3], today braces are quite different in design, and possibly in terms of efficacy [4]. Not all braces recognize in their design the same biomechanical actions as demonstrated by many papers [22, 25–27]. From a biomechanical point of view, the stiffness coefficient of the material is one of the characteristics of the brace, but it’s not enough to achieve good results. For example, a plaster brace, using the same material, can be more or less effective, depending on its final geometry and how the corrective forces are applied during the packaging. These affect the interactions between the plaster and trunk of the subject.

**Fig. 5:**
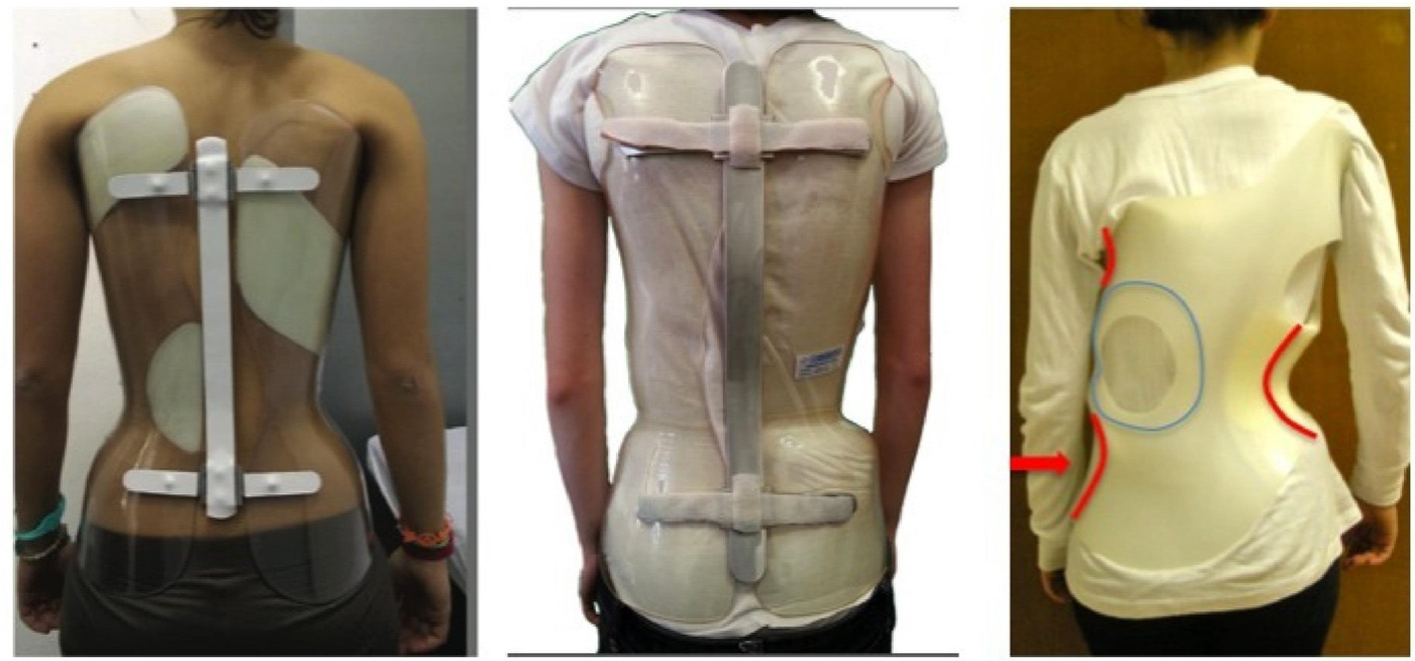
SPoRT (Sforzesco) Brace (*left*), ART Brace (*middle*), Rigo-Cheneau Brace (*Right*). The differences in the level of symmetry and asymmetry is evident

The new super-rigid braces, which have been created to treat high-degree curves, showed interesting results, with the Sforzesco already tested even in surgical curves [20], while the ART, being born more recently, has been tested in a wider range of curves [22]. For this reason, we chose for the comparison only curves larger than 40°, for which the risk of progression to the surgical threshold according to the natural history is extremely high. Moreover, this is the only indication in the SPoRT (Sforzesco brace) database that includes all patients, while in less important curves other braces are used too. This limited the availability of patients, resulting in a small sample size. This is a limitation of the study; nevertheless, these preliminary results are really interesting. In fact, we have found that the marked asymmetry of the envelope could possibly be useful for reaching a slightly better in-brace correction, but this difference is not statistically significant nor clinically relevant, and it is, in any case, reduced in short-term out-of-brace comparison. Some of the most frequently used modern braces, such as the Rigo-Cheneau brace, base their action on the marked envelope asymmetry that warrants an asymmetrical in-brace posture to revert the curve [27]. In contrast, the SPoRT concept of bracing is based on an almost symmetric envelope with pushes acting inside the brace to exert higher forces on the trunk and creating a lower degree of asymmetry [17]. The ART brace mixed both the concepts of rigidity and asymmetry, adopting the same material as the SPoRT brace and an asymmetric envelope. It could be argued that the asymmetry of the ART brace is not so important (Fig. 5), since there are much more asymmetric braces [27], but asymmetry remains probably the main difference between the two braces. Moreover, this was a very specific comparison of the ART and SPoRT (Sforzesco) braces, and we are not confident in generalizing these results to different kinds of braces.

In-brace correction has always been considered a key element of scoliosis treatment, with studies demonstrating that a high percentage of correction is needed to achieve good results. These studies have been performed in lower-degree curves, up to 30–35°, while our curves are much larger, up to 60° Cobb, and a percentage comparison is not correct, as larger curves have higher rigidity and presumably reduced in-brace correction. Nevertheless, it’s interesting to note that an overall good correction, even if lower than in other cases, didn’t affect at least 6-months results. We can assume that at least for super-rigid braces the in-brace correction is only partially predictive, as already shown [28]. Since this study considered only major curves, in the future it could be appropriate to also assess the value of rotations, which has been considered by some authors as the largest index of structuration [29]. Rotation could be a predictive criterion for treatment failure: in fact, Aulisa showed that in curves with rotation of more than 20° measured according to Perdriolle, it was not possible to respond to any corrective action applying the PASB brace in curves larger than 45° [29]. The anatomical modifications of the disc, caused by a permanent rotation greater than 20 Perdriolle degrees, are characterized by hysteresis of the fibers of the annulus. This leads to an increase of the module of the torsional stiffness and this could make the disc indifferent to the derotation action of any type of brace, but we don't have enough data to be sure about and it is possible that the super-rigid material could overcome this limit.

The main comparisons were made using a parametric test. This can be a limitation for an immediate clinical generalizability of results. But we chose not to categorize results using the traditional 5° Cobb threshold in order to increase the power of the statistical analysis and discover even small differences. This was important since we couldn’t perform a sample size calculation as no other similar studies were available. For the same reason, we included some patients with Risser 4 in both groups and the overall population was quite mature. Even if these patients have a really limited progression risk, it’s still interesting to report that they can be improved. This is relevant and helpful if we think that all the patients had high-degree curves, and an improvement can reduce the risk of future surgery. These findings, moreover, are consistent with previous reports made on patients starting treatment very late at Risser 4–5, with 46 % of patients with curves larger than 30° being improved at the end of treatment [30].

According to the SOSORT and SRS recommendations for research studies on the treatment of idiopathic scoliosis, new braces can be compared in the very short-term considering the in-brace correction, and at short-term at one year, with results that are considered preliminary [12]. We respected this as we used the in-brace correction, but we haven’t been able to compare data at one year since the clinical protocols of both centers program the first radiography at 6 months from the beginning of bracing, and then after one year more. Due to the lack of direct comparison of braces, and with one of the two being very new, this is appropriate and helpful for clinicians, with further studies to be performed for confirmation.

The Cobb angle and the progression/improvement rate are really important and useful for scoliosis patients. Nevertheless, other outcomes such as quality of life and aesthetic improvements are even more relevant [6]. In this study we could not assess them since these data were collected only from some of the patients and this is a limitation, but future studies should do so, together with a pre/post evaluation of the sagittal profile, which is even more important for adulthood stability and as a predictor of back pain [31, 32].

## Conclusion

Radiographic results in the very short and short term were similar for the ART and Sforzesco braces. Further studies with end growth results are needed to confirm these preliminary data. Sagittal profile assessment, aesthetic changes, vertebral rotation, compliance and quality of life measurement should be included for a more complete and patient-oriented evaluation of the treatment effects.

## Additional files

Additional file 1:
**Questionnaire to verify the achievement of the SOSORT Criteria for bracing: “Standards of management of idiopathic scoliosis with corrective braces in everyday clinics and in clinical research” - Filled by Fabio Zaina for the Sforzesco group (ISICO).**
Additional file 2:
**Questionnaire to verify the achievement of the SOSORT Criteria for bracing: “Standards of management of idiopathic scoliosis with corrective braces in everyday clinics and in clinical research” Filled by Jean Claude de Mauroy for ART group.**

## References

[1] Weinstein SL, Dolan LA, Wright JG, Dobbs MB (2013). Effects of Bracing in Adolescents with Idiopathic Scoliosis. N Engl J Med.

[2] Coillard C, Circo AB, Rivard CH (2014). A Prospective Randomized Controlled Trial of the Natural History of Idiopathic Scoliosis versus treatment with the Spinecor brace. Sosort Award 2011 Winner. Eur J Phys Rehabil Med.

[3] Rigo M, Negrini S, Weiss HR, Grivas TB, Maruyama T, Kotwicki T (2006). “SOSORT consensus paper on brace action: TLSO biomechanics of correction (investigating the rationale for force vector selection)”. Scoliosis.

[4] Zaina F, De Mauroy JC, Grivas T, Hresko MT, Kotwizki T, Maruyama T (2014). Bracing for scoliosis in 2014: state of the art. Eur J Phys Rehabil Med.

[5] Negrini S, Grivas TB, Kotwicki T, Rigo M, Zaina F, international Society on Scoliosis Orthopaedic and Rehabilitation Treatment (SOSORT) (2009). Guidelines on “Standards of management of idiopathic scoliosis with corrective braces in everyday clinics and in clinical research”: SOSORT Consensus 2008. Scoliosis.

[6] Negrini S, Minozzi S, Bettany-Saltikov J, Zaina F, Chockalingam N, Grivas TB (2010). Braces for idiopathic scoliosis in adolescents. Cochrane Database Syst Rev.

[7] Richards BS, Bernstein RM, D’Amato CR, Thompson GH (2005). Standardization of criteria for adolescent idiopathic scoliosis brace studies: SRS Committee on Bracing and Nonoperative Management. Spine.

[8] Janicki JA, Poe-Kochert C, Armstrong DG, Thompson GH (2007). A comparison of the thoracolumbosacral orthoses and providence orthosis in the treatment of adolescent idiopathic scoliosis: results using the new SRS inclusion and assessment criteria for bracing studies. J Pediatr Orthop.

[9] Shaughnessy WJ (2007). Advances in scoliosis brace treatment for adolescent idiopathic scoliosis. Orthop Clin North Am.

[10] Negrini S, Donzelli S, Lusini M, Minnella S, Zaina F (2014). The effectiveness of combined bracing and exercise in adolescent idiopathic scoliosis based on SRS and SOSORT criteria: a prospective study. BMC Musculoskelet Disord.

[11] Aulisa AG, Guzzanti V, Marzetti E, Giordano M, Falciglia F, Aulisa L (2014). Brace treatment in juvenile idiopathic scoliosis: a prospective study in accordance with the SRS criteria for bracing studies-SOSORT award 2013 winner. Scoliosis.

[12] Negrini S, Hresko TM, O’Brien JP, Price N, SOSORT Boards, SRS Non-Operative Committee (2015). Recommendations for research studies on treatment of idiopathic scoliosis: Consensus 2014 between SOSORT and SRS non-operative management committee. Scoliosis.

[13] Negrini S, Aulisa AG, Aulisa L, Circo AB, De Mauroy JC, Durmala J (2012). 2011 SOSORT guidelines: Orthopaedic and Rehabilitation treatment of idiopathic scoliosis during growth. Scoliosis.

[14] Fayssoux RS, Cho RH, Herman MJ (2010). A history of bracing for idiopathic scoliosis in North America. Clin Orthop.

[15] Morin C, Kulkarni S (2014). ED plaster-of-Paris jacket for infantile scoliosis. Eur Spine J Off Publ Eur Spine Soc Eur Spinal Deform Soc Eur Sect Cerv Spine Res Soc.

[16] Fletcher ND, McClung A, Rathjen KE, Denning JR, Browne R, Johnston CE (2012). Serial casting as a delay tactic in the treatment of moderate-to-severe early-onset scoliosis. J Pediatr Orthop.

[17] Negrini S, Marchini G, Tessadri F (2011). Brace technology thematic series-The Sforzesco and Sibilla braces, and the SPoRT (Symmetric, Patient oriented, Rigid, Three-dimensional, active) concept. Scoliosis.

[18] Negrini S, Marchini G (2007). Efficacy of the symmetric, patient-oriented, rigid, three-dimensional, active (SPoRT) concept of bracing for scoliosis: a prospective study of the Sforzesco versus Lyon brace. Eur Medicophysica.

[19] Negrini S, Atanasio S, Negrini F, Zaina F, Marchini G (2008). The Sforzesco brace can replace cast in the correction of adolescent idiopathic scoliosis: A controlled prospective cohort study. Scoliosis.

[20] Lusini M, Donzelli S, Minnella S, Zaina F, Negrini S. Brace treatment is effective in idiopathic scoliosis over 45°: an observational prospective cohort controlled study. Spine J. 2013. 10.1016/j.spinee.2013.11.040 24295798

[21] De Mauroy JC, Lecante C, Barral F (2011). “Brace Technology” Thematic Series-The Lyon approach to the conservative treatment of scoliosis. Scoliosis.

[22] De Mauroy JC, Lecante C, Barral F, Pourret S (2014). Prospective study and new concepts based on scoliosis detorsion of the first 225 early in-brace radiological results with the new Lyon brace: ARTbrace. Scoliosis.

[23] Bunnell WP (1984). An objective criterion for scoliosis screening. J Bone Joint Surg Am.

[24] Hacquebord JH, Leopold SS (2012). In Brief: The Risser Classification: A Classic Tool for the Clinician Treating Adolescent Idiopathic Scoliosis. Clin Orthop Relat Res.

[25] Coillard C, Leroux MA, Zabjek KF, Rivard CH (2003). SpineCor--a non-rigid brace for the treatment of idiopathic scoliosis: post-treatment results. Eur Spine J Off Publ Eur Spine Soc Eur Spinal Deform Soc Eur Sect Cerv Spine Res Soc.

[26] Aulisa AG, Mastantuoni G, Laineri M, Falciglia F, Giordano M, Marzetti E (2012). Brace technology thematic series: the progressive action short brace (PASB). Scoliosis.

[27] Rigo MD, Villagrasa M, Gallo D (2010). A specific scoliosis classification correlating with brace treatment: description and reliability. Scoliosis.

[28] Zaina F, Donzelli S, Lusini M, Negrini S (2012). Correlation between in-brace radiographic correction and short time brace results. Stud Health Technol Inform.

[29] Aulisa AG, Guzzanti V, Giordano M, Falciglia F, Fuiano M, Aulisa L (2014). Conservative treatment in adolescent idiopathic scoliosis with curves over 45°: is the measurement in Cobb degrees the only parameter to be considered?. Scoliosis.

[30] Negrini S, Donzelli S, Lusini M, Zaina F (2012). Bracing can reduce high degree curves and improve aesthetics immediately after the end of growth. Final results of a retrospective case series. Stud Health Technol Inform.

[31] Glassman SD, Bridwell K, Dimar JR, Horton W, Berven S, Schwab F (2005). The impact of positive sagittal balance in adult spinal deformity. Spine.

[32] Schwab F, Ungar B, Blondel B, Buchowski J, Coe J, Deinlein D (2012). Scoliosis Research Society-Schwab adult spinal deformity classification: a validation study. Spine.

