# Supplementary material for: SOSORT Award Winner 2015: a multicentre study comparing the SPoRT and ART braces effectiveness according to the SOSORT-SRS recommendations
Source: Scoliosis. 2015 Aug 11;10:23. doi: 10.1186/s13013-015-0049-4 (PMC4532257; doi:10.1186/s13013-015-0049-4)
Supplement: Additional file 1: — Questionnaire to verify the achievement of the SOSORT Criteria for bracing: “Standards of management of idiopathic scoliosis with corrective braces in everyday clinics and in clinical research” - Filled by Fabio Zaina for the Sforzesco group (ISICO). [file 13013_2015_49_MOESM1_ESM.doc]

# Questionnaire to verify the achievement of the SOSORT Criteria for bracing: “Standards of management of idiopathic scoliosis with corrective braces in everyday clinics and in clinical research” – Filled by Fabio Zaina for the Sforzesco group (ISICO)

This questionnaire has been developed to

- allow each professional to self-test if he satisfies recommended management criteria for bracing
- test in case of research studies if the management of patients has been adequate according to the actual standards
- help patients understand if their caregivers satisfy the actual management needs

Ideally all the answers to the questions should be “Yes”.

During the SOSORT Consensus, cumulative answers in terms of clinical behaviors were:

- 38% no negative answers
- 53% up to 1 negative answer
- 68% up to 5 negative answers
- 91% up to 8 negative answers

Consequently, provided all 44 answers are given (if it lacks one member of the team, all relative answers should be “no”), until new researches will refine the system, we propose

- Excellent: 0-1 no out of 44
- Good: 2-5 no out of 44
- Sufficient: 6-8 no out of 44
- Insufficient: 9 no or more out of 44

We are aware that these standards are not applicable everywhere in the world, currently, for many different reasons. Nevertheless, we strongly support their progressive application, and SOSORT is ready to support individuals and groups who need help in reaching these minimum standards through education and masterships.

For more information look at the journal Scoliosis ([www.scoliosisjournal.com](http://www.scoliosisjournal.com/)) where the original paper (Negrini S, Grivas TB, Kotwicki T, Rigo M, Zaina F. “Standards of management of idiopathic scoliosis with corrective braces in everyday clinics and in clinical research. SOSORT Consensus 2008”) has been published in 2008, and to the SOSORT web site ([www.sosort.org](http://www.sosort.org/)).

# All professionals as a team

Do you work in a multiprofessional team (physician, orthotist and eventually physiotherapist), through continuous exchange of information, team meetings, and verification of braces in front of single patients? ~~No~~ Yes

Do you give thorough advice and counselling to each single patient and family each time it is needed? ~~No~~ Yes

Do the different professionals in your team give the same, previously agreed messages to patients and families? ~~No~~ Yes

Do you check each single brace in team (physician, orthotist, and possibly physiotherapist)? ~~No~~ Yes

Do you follow-up regularly each single brace? ~~No~~ Yes

Do you access the patient’s mood and counsel him and the family at brace delivery and at other follow-ups? ~~No~~ Yes

Do you check each single brace clinically and/or radiographically? ~~No~~ Yes

Do you check the brace and patient compliance regularly and reinforce the usefulness of brace treatment to the patient and his/her family? ~~No~~ Yes

# Medical Doctors

Have you been trained by a previous master (i.e. a physician with at least 5 years of experience in bracing) for at least 2 years? ~~No~~ Yes

Did you have at least 2 years of continuous practice in scoliosis bracing? ~~No~~ Yes

Have you prescribed at least 1 brace per working week (~45 per year) in the last 2 years? ~~No~~ Yes

Have you evaluated at least 4 scoliosis patients per working week (~150 per year) in the last 2 years? ~~No~~ Yes

Do you prescribe each single brace to the constructing orthothist? ~~No~~ Yes

Do you write the details of brace construction (where to push and where to leave space, how to act on the trunk to obtain results on the spine) when not already defined “a priori” with the orthotist? ~~No~~ Yes

Do you prescribe the exact number of hours of brace wearing? ~~No~~ Yes

Are you totally convinced of the brace proposed and committed to the treatment? ~~No~~ Yes

Do you use any ethical mean to increase patient compliance, including thorough explanation of the treatment, aids such as photos, brochures, video, etc? ~~No~~ Yes

Do you verify accurately if the brace fits properly and fulfils the need of the individual patient? ~~No~~ Yes

Do you check the scoliosis correction in all the three planes (frontal, sagittal and horizontal)? ~~No~~ Yes

Do you check clinically the aesthetic correction? ~~No~~ Yes

Do you maximize brace tolerability (reduce visibility and allow movements and activity of daily life as much as possible for the used technique)? ~~No~~ Yes

Do you check the corrections applied? ~~No~~ Yes

Do you follow-up the braced patients regularly, at least every 3 to 6 months? ~~No~~ Yes

Do you reduce standard intervals according to individual needs (first brace, growth spurt, progressive or atypical curve, poor compliance, request of other team members)? ~~No~~ Yes

Do you take the responsibility to change the brace for a new one as soon as the child grows up or the brace loses efficacy? ~~No~~ Yes

# Orthotists

Have you been working continuously with a master physician (i.e. a physician fulfilling to recommendation 1 criteria) for at least 2 years? ~~No~~ Yes

Did you have at least 2 years of continuous practice in scoliosis bracing? ~~No~~ Yes

Have you constructed at least 2 braces per working week (~100 per year) in the last 2 years? ~~No~~ Yes

Do you construct each single brace according to physician prescription? ~~No~~ Yes

Do you correct each single brace according to physician indications? ~~No~~ Yes

Do you check the prescription and its details and eventually discuss them with the prescribing physician, if needed, before construction? ~~No~~ Yes

Do you fully execute the agreed prescription? ~~No~~ Yes

Are you totally convinced of the brace proposed and committed to the treatment? ~~No~~ Yes

Do you use any ethical mean to increase patient compliance, including thorough explanation of the treatment, aids such as photos, brochures, video, etc? ~~No~~ Yes

Do you maximize brace tolerability (reduce visibility and allow movements and activity of daily life as much as possible for the used technique)? ~~No~~ Yes

Do you apply all changes needed and, if necessary, even rebuild the brace without extra-charge for patients? ~~No~~ Yes

Do you suggest to change the brace for a new one as soon as the child grows up or the brace loses efficacy? ~~No~~ Yes

Do you check regularly the brace ? ~~No~~ Yes

In front of any problem with the brace, do you refer to the treating physician? ~~No~~ Yes

# Physiotherapists

Do you check the brace when you evaluate/treat a patient wearing a brace? No ~~Yes~~

In front of any problem with the brace, do you refer to the treating physician? ~~No~~ Yes

In front of any problem with the brace, do you avoid to refer to the patient? ~~No~~ Yes

If you are a member of the treating team, have you been trained to face the problems of compliance, and the needs of explanation by the patient or his/her family? ~~No~~ Yes

If you are not a member of the treating team, do you avoid acting autonomously? ~~No~~ Yes
